# A Rare Case of the Coexistence of Pancreaticobiliary Maljunction and Gastrointestinal Tumor in Neurofibromatosis Type 1

**DOI:** 10.7759/cureus.24048

**Published:** 2022-04-11

**Authors:** Rie Tanaka, Akinori Sekioka, Shuichi Ota, Tetsuo Ito, Yukito Adachi

**Affiliations:** 1 Gastroenterological Surgery, Osaka Saiseikai Noe Hospital, Osaka, JPN

**Keywords:** case report, intrahepatic bile duct, pancreaticobiliary maljunction, gastrointestinal stromal tumor, neurofibromatosis type 1

## Abstract

Neurofibromatosis type 1 (NF1) is a congenital condition characterized by "café au lait" spots and subcutaneous fibromas. There are various combined diseases, such as malignant tumors in the abdominal organs or brain tumors.

Here, we present a case of a 35-year-old patient with a rare combination of NF1 with a gastrointestinal stromal tumor (GIST) and pancreaticobiliary maljunction (PBM). At the first visit, her main symptom was right upper abdominal pain. Radiological investigations revealed a common bile duct stone, submucosal tumor in the duodenum, PBM, and abnormal findings in the intrahepatic bile ducts. After the common bile duct stone was removed by endoscopic intervention, the patient underwent laparoscopic cholecystectomy, resection of the duodenal submucosal tumor, and liver biopsy. Pathological examination revealed chronic cholecystitis, GIST of the duodenum, and chronic inflammation of the intrahepatic bile ducts.

This is the first case report of the rare coexistence of GIST and PBM in a patient with NF1.

## Introduction

Neurofibromatosis type 1 (NF1) is a rare hereditary disease caused by alterations in the NF1 gene [1]. Because of the high mutation rates of NF1, approximately half of the patients had no family history of the disorder [1]. NF1 often occurs in combination with other conditions, such as benign or malignant tumors. Since some of the combined diseases can be fatal, regular checkups are recommended for patients with NF1 [2].

Gastrointestinal stromal tumors (GISTs) are uncommon neoplasms derived from Cajal’s interstitial cells in the gastrointestinal tract [3]. The incidence of GISTs ranges from 10 to 15 per million per year, which are commonly found in the stomach, whereas approximately 5%-25% of patients with NF1 have GISTs that are mostly found in the small intestine [4, 5].

Pancreaticobiliary maljunction (PBM) is a rare congenital anomaly, defined as the union of the pancreatic and biliary ducts located outside the duodenal wall [6]. Although the majority of cases of PBM are in conjunction with biliary duct dilatation, only a few of them are not combined with biliary duct dilatation. The problem with PBM is an increase in the carcinogenic rate of the gallbladder and bile duct. PBM with bile duct dilatation is known to develop into bile duct carcinoma, whereas 36.1% of PBMs without bile duct dilatation develop into gallbladder carcinoma [6].

In this report, we present a rare case of the coexistence of duodenal GIST and PBM in a patient with NF1.

## Case presentation

**Figure 1:**
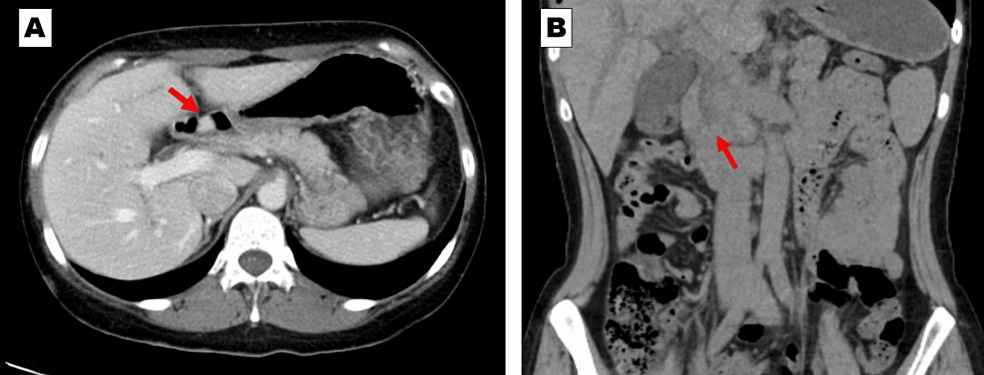
Abdominal computed tomography Abdominal computed tomography reveals a stone in the lower common bile duct and a tumor in the duodenal bulb. A: 8×9-mm-size tumor in the duodenal bulb (arrow). B: The common bile duct stone (arrow).

**Figure 2:**
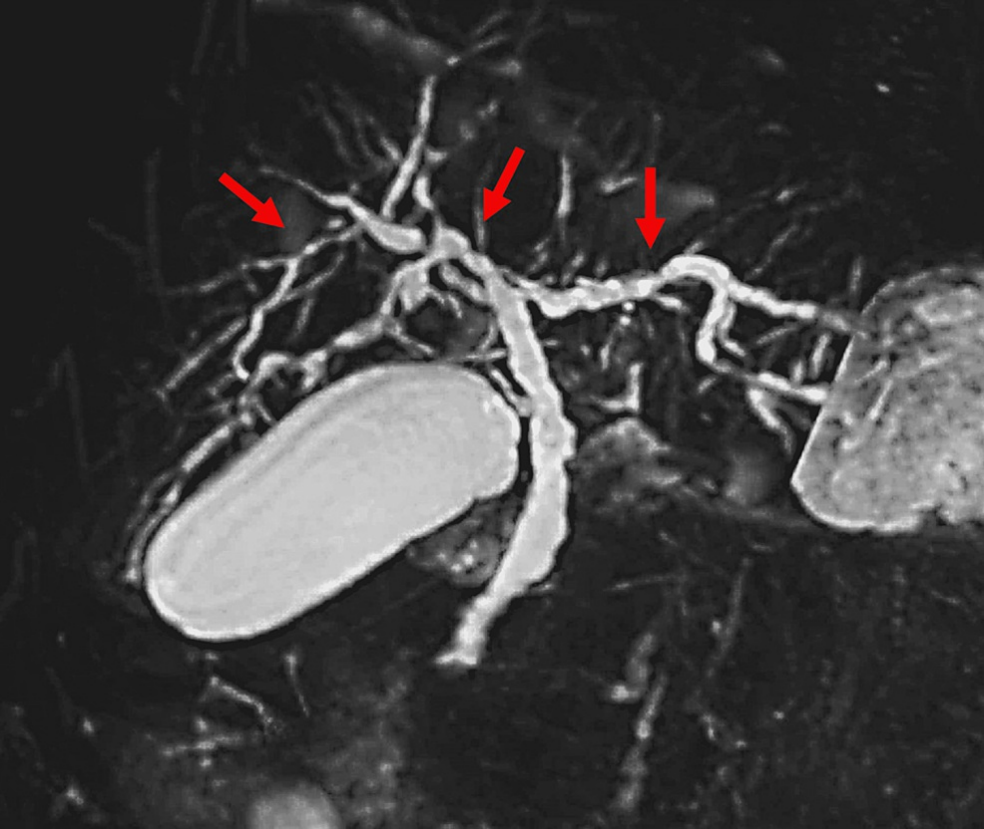
Magnetic resonance cholangiopancreatography Magnetic resonance cholangiopancreatography shows diffuse stenosis and dilation of the intrahepatic and extrahepatic bile ducts (arrows).

**Figure 3:**
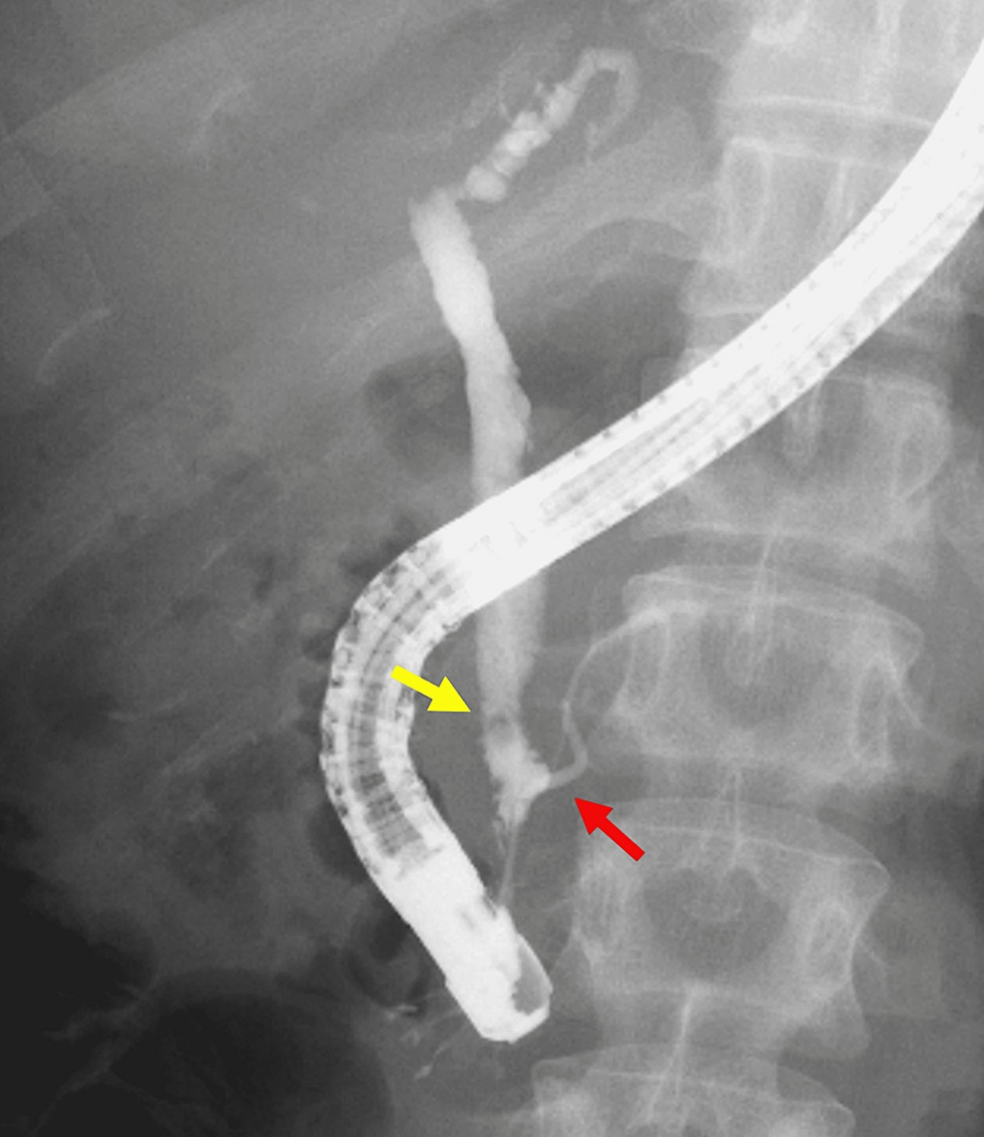
Endoscopic retrograde cholangiopancreatography Endoscopic retrograde cholangiopancreatography reveals the junction of the pancreatic duct and bile duct located outside the duodenal wall with a long common channel (red arrow). The yellow arrow shows the filling defect of the common bile duct consistent with the stone.

**Figure 4:**
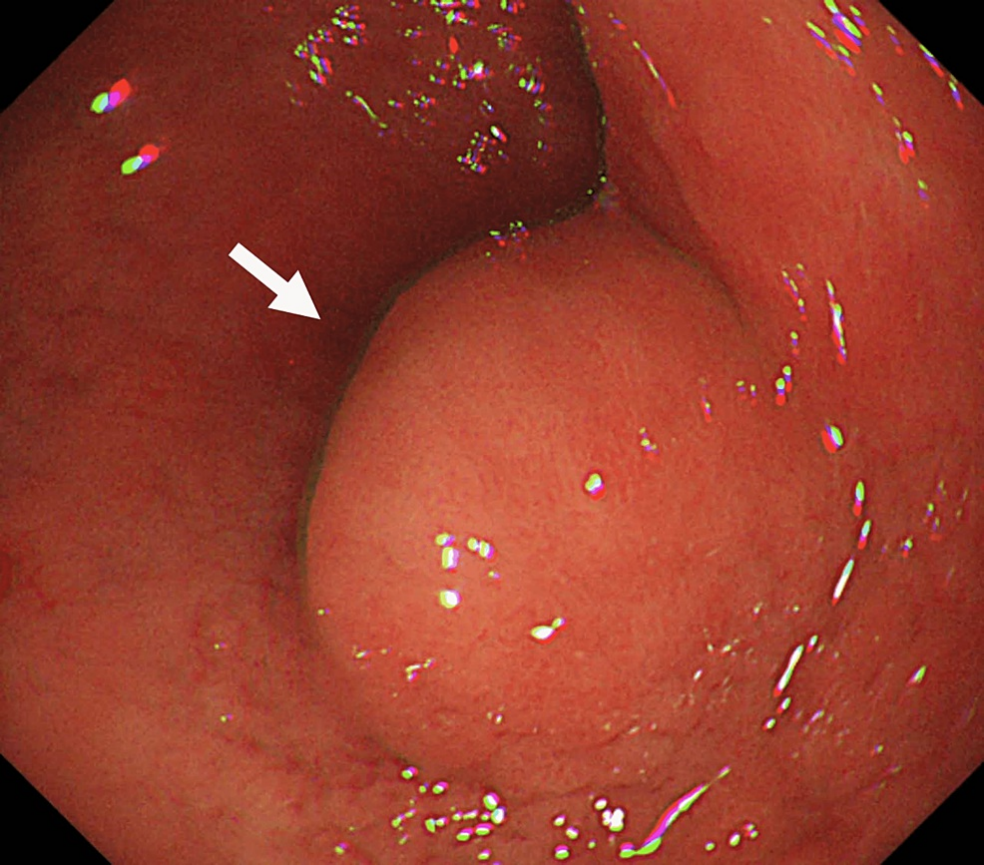
Esophagogastroduodenoscopy Esophagogastroduodenoscopy reveals a submucosal tumor on the anterior wall of the duodenal bulb (arrow).

A 35-year-old woman with NF1 was referred to the emergency care department of our hospital with intermittent severe epigastric pain. Abdominal contrast-enhanced computed tomography revealed a stone in the lower common bile duct and a tumor in the duodenal bulb (Figure 1). Magnetic resonance cholangiopancreatography (MRCP) revealed irregular walls of the intrahepatic and extrahepatic bile ducts, indicating chronic inflammation (Figure 2). Endoscopic retrograde cholangiopancreatography revealed a common bile duct stone and PBM with no biliary duct dilatation (Figure 3). After the common bile duct stone was removed, abdominal pain improved. The amylase level of the bile was 9,009 (reference range, <1,000) IU/L, and cytologic examination of the bile was reported as suspicious for malignancy. Esophagogastroduodenoscopy revealed a submucosal tumor on the anterior wall of the duodenal bulb (Figure 4). Her blood test (seven months after the removal of the common bile duct stone) showed high levels of alkaline phosphatase (522 [reference range, 38-113] IU/L), alanine transaminase (142 [reference range, 5-45] IU/L), and gamma-glutamyltransferase (288 [reference range, 0-48] IU/L). The levels of carcinoembryonic antigen (2.2 [reference range, 0.1-5.0] ng/mL), carbohydrate antigen 19-9 (21.0 [reference range, <37] U/mL), and immunoglobulin G (1,186 [reference range, 870-1,700] mg/dL) were normal.

**Figure 5:**
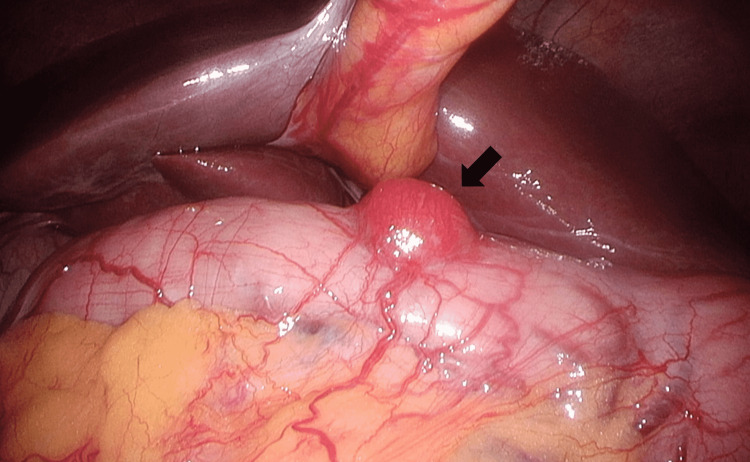
Laparoscopic image The tumor on the anterior wall of the duodenal bulb is identified during the surgery (arrow). The tumor appeared to be intramural.

**Figure 6:**
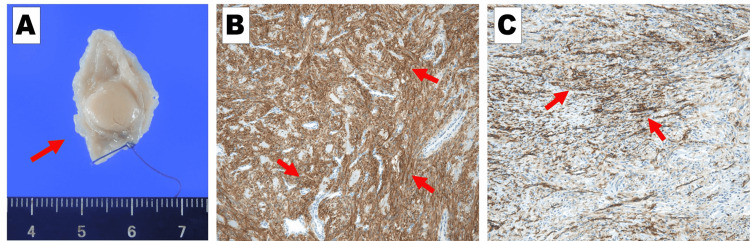
Histopathological findings of the gastrointestinal stromal tumor The gastrointestinal stromal tumor is 9×8 mm in size. Immunohistochemistry shows that c-KIT(CD117) and CD34 are both positive. A: Macroscopic image of the tumor (arrow). B: Immunohistochemical c-KIT(CD117) expression. Positive cells are stained brown (arrows). C: Immunohistochemical CD34 expression. Positive cells are stained brown (arrows).

**Figure 7:**
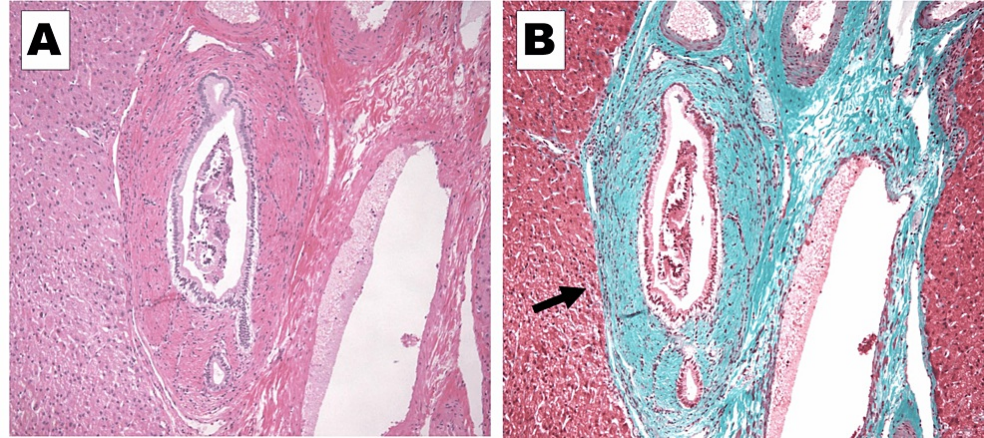
Histopathological findings of the liver Histopathological findings of the liver show periductal fibrosis. A: Hematoxylin and Eosin staining. B: Masson’s trichrome staining. Blue staining (arrow) shows collagen accumulation.

Based on these results, laparoscopic cholecystectomy, partial duodenal resection, and liver biopsy were performed. A tumor on the anterior wall of the duodenal bulb was identified during the surgery (Figure 5). We placed two stay sutures cranial and caudal to the resection site, and resected the tumor using the endovascular gastrointestinal anastomosis stapler. Histopathological results showed chronic cholecystitis (negative for malignancy), GIST of the duodenum (Figure 6), chronic inflammation of the intrahepatic bile ducts, and periductal fibrosis, indicating the possibility of primary sclerosing cholangitis (PSC) (Figure 7). The duodenal GIST was positive for c-KIT(CD117) and CD34 and negative for Smooth muscle alpha-actin and S100. The Ki-67 labeling index was 1.6%. The tumor was 0.9 cm in size, and mitotic activity was very low (1/50 high-power field). Based on these results, the tumor was classified as very low risk according to the National Institutes of Health criteria and as category 1 according to the Armed Forces Institute of Pathology criteria. There were no postoperative complications, and the patient was discharged six days postoperatively. Clinical follow-up at one year showed no recurrence of GIST, and the irregular walls of the intrahepatic bile ducts remained the same.

## Discussion

NF1, historically known as von Recklinghausen’s disease, is an autosomal dominant hereditary syndrome usually diagnosed in childhood or adolescence [5]. NF1 is diagnosed by the presence of two or more of the major clinical criteria established by the National Institutes of Health Consensus Development Conference [1,7]. Most gastrointestinal manifestations of NF1 arise during midlife or later [1]. This patient was also diagnosed with NF1 in early childhood from multiple café au lait macules and neurofibromas, and to the best of our knowledge, this is the first report of gastrointestinal abnormality.

The most common tumor affecting the gastrointestinal tract in patients with NF1 is GIST [5]; however, only 5% of patients have symptoms [1]. GISTs in NF1 are usually multiple, small, and mitotically inactive. Patients with a tumor size of >5 cm and brisk mitotic activity are at risk of poor outcomes [8]. Surgical management is the standard treatment for GISTs [3]. In this case, there were no symptoms related to the submucosal tumor; however, it was found incidentally during the examination. Although the tumor was small, approximately 1 cm in size, and the pathological findings were unclear before surgery, we planned to resect the tumor simultaneously with cholecystectomy because NF1 is often combined with GIST.

PBM is an abnormal junction of the pancreaticobiliary ducts that leads to the reflux of bile and pancreatic juice. Because of this reflux, it is known to cause cancers of the bile duct or gallbladder [6]. PBM with biliary duct dilatation is associated with a high risk of bile duct carcinoma. On the other hand, PBM without biliary duct dilatation is associated with a high risk of gallbladder carcinoma. Therefore, in the latter situation, cholecystectomy is recommended to prevent gallbladder cancer [9]. In this case, there was no dilatation in the biliary duct; therefore, cholecystectomy was performed.

In the present case, MRCP showed irregular shapes and walls of the intrahepatic bile ducts; therefore, a liver biopsy was planned to clarify the underlying disease. There were several possible diseases, such as PSC, IgG4-related hepatitis, and secondary changes due to PBM. The patient’s history and blood test and liver biopsy results did not confirm the diagnosis. Certainly, the bile duct appearance on MRCP suggested the possibility of PSC, but the elevation of serum alkaline phosphatase level was transient, and there were no characteristic findings of PSC in the liver biopsy specimen. According to the diagnostic criteria of PSC, this patient was diagnosed with probable PSC [10]. Either way, the existence of irregular walls of the intrahepatic bile duct is associated with a high risk of intrahepatic stones; therefore, regular follow-up is needed in the future.

The coexistence of NF1 and PBM is rare. NF1 and PBM are both congenital diseases, but the hereditary cause of PBM is unclear. To the best of our knowledge, only one previous report has shown a combination of neuroendocrine tumor (NET) and PBM with biliary duct dilatation [11]. There are no case reports of the coexistence of GIST and PBM in patients with NF1. In the present case, these rare conditions coexisted, but adequate examination and accurate diagnosis led to proper treatment of the patient in one surgery.

## Conclusions

We present a rare case of the coexistence of duodenal GIST and PBM in a patient with NF1. We were able to provide proper treatment through preoperative examination and consideration of the patient’s background. When seeing an NF1 patient, we should always consider the possibility of a hidden tumor, as well as any abnormal structures in the bile duct. Since the relevance of NF1 and PBM is unclear, further research would be warranted.
